# Polymer–Nickel Composite Filaments for 3D Printing of Open Porous Materials

**DOI:** 10.3390/ma15041360

**Published:** 2022-02-12

**Authors:** Ewelina Mackiewicz, Tomasz Wejrzanowski, Bogusława Adamczyk-Cieślak, Graeme J. Oliver

**Affiliations:** 1 Division of Materials Design, Faculty of Materials Science and Engineering, Warsaw University of Technology, Woloska 141, 02-507 Warsaw, Poland; boguslawa.cieslak@pw.edu.pl; 2 Department of Mechanical Engineering, Cape Peninsula University of Technology, P.O. Box 1906, Bellville 7535, South Africa; oliverg@cput.ac.za

**Keywords:** composites, polymers, additive manufacturing, porous materials, Fused Deposition Modeling

## Abstract

Catalysis has been a key way of improving the efficiency-to-cost ratio of chemical and electrochemical processes. There have been recent developments in catalyst materials that enable the development of novel and more sophisticated devices that, for example, can be used in applications, such as membranes, batteries or fuel cells. Since catalytic reactions occur on the surface, most catalyst materials are based on open porous structures, which facilitates the transport of fluids (gas or liquid) and chemical (or electrochemical) specific surface activity, thus determining the overall efficiency of the device. Noble metals are typically used for low temperature catalysis, whereas lower cost materials, such as nickel, are used for catalysis at elevated temperatures. 3D printing has the potential to produce a more sophisticated fit for purpose catalyst material. This article presents the development, fabrication and performance comparison of three thermoplastic composites where PLA (polylactic acid), PVB (polyvinyl butyral) or ABS (acrylonitrile butadiene styrene) were used as the matrix, and nickel particles were used as filler with various volume fractions, from 5 to 25 vol%. The polymer–metal composites were extruded in the form of filaments and then used for 3D FDM (Fused Deposition Modeling) printing. The 3D printed composites were heat treated to remove the polymer and sinter the nickel particles. 3D printed composites were also prepared using nickel foam as a substrate to increase the final porosity and mechanical strength of the material. The result of the study demonstrates the ability of the optimized filament materials to be used in the fabrication of high open porosity (over 60%) structures that could be used in high-temperature catalysis and/or electrocatalysis.

## 1. Introduction

Three-dimensional (3D) printing is also known as rapid prototyping or additive manufacturing (AM) of material structures directly from digital models developed with the use of computer aided design (CAD) software. Therefore, this technology is classified as a ‘bottom-up’ approach and it is based on the incremental addition of material layers leading to near net-shape realization of 3D designs. Common additive manufacturing technologies include: Fused Deposition Modeling (FDM), Stereolithography (SLA), Direct Ink Writing (DIW), Selective Laser Sintering (SLS) and Selective Laser Melting (SLM) [1,2]. The methods have interesting scientific and engineering potential in that they allow one to use a variety of methods for fabrication combining polymers, metals or ceramics in the form of filaments or powders (also combined). 3D printing thus offers new possibilities particularly in the more accurate tuning of the structure of functional materials, but also in modifying their chemical composition [3,4], as compared with traditional methods. Therefore, it is seemingly the most advantageous technique for the manufacturing of porous materials, which are used as heterogeneous catalysts or catalyst supports [5,6,7,8,9,10,11] and biomedical implants or scaffolds [12,13,14], but also filters, separators, membranes or supercapacitors [15,16,17,18]. 

3D printing technology is being broadly applied in the manufacturing of 3D materials for catalysis [19,20,21], especially for open porous catalysts and their supports. These methods allow one to use different materials to realize unique, complex—random or ordered—spatial structures with sophisticatedly designed characteristics, such as specific porosity and mean pore size [20]. Secondly, it offers great dimensional repeatability and reproducibility in the fabrication of structures since they are based on digital models acting as templates in the fabrication process. Various materials have been used to date to manufacture such porous structures, e.g., thermoplastic polymers (acrylonitrile butadiene styrene—ABS, polylactic acid—PLA and high impact polystyrene—HIPS), non-metals (carbon), metals (iron, nickel, cobalt and ruthenium) or ceramics (TiO_2_, Al_2_O_3_, and ZnO). In some systems, the catalytic activity is determined by the inherent properties of the support material, while in other systems, it is determined from the interaction between the support and catalyst present on the surface of the support [22,23,24]. Some other approaches consist of incorporating catalyst particles into polymeric precursors (e.g., filaments) to form inorganic/organic hybrid materials. Examples might be hybrid filaments made of BaTiO_3_/ABS [25], TiO_2_/ABS [26], Fe/ABS [27], Fe_3_O_4_/polycaprolactone [28] or graphene/ABS [29]. The crucial aspect is the amount of a given additive since it may negatively affect the rheological characteristics of the feedstock.

New composite filaments with catalytically active powders embedded into a thermally or chemically removable polymeric matrix opens up a wide range of applications in chemistry and electrochemistry [1,6,7]. Those two different applications require slightly different types of porous structures. For chemical catalysis, the highest specific surface area is of key importance, while electrochemical processes require the material to be electrically conductive, which imposes a reduction of the specific surface due to the need for powder particles to be better connected (highly sintered). Both applications demand the highest specific catalytic activity, which is mostly dependent on the chemical composition of the powders.

Open porous materials are used for catalysis because of the advantages they offer in terms of facilitating the transport of reactants and the removal of products as well as offering a large surface area or density of active sites for chemical reactions to take place. Porosity and mean pore size are the key parameters in terms of having sufficient transport of gaseous or liquid media (reactants), desorption and the removal of products into the pore space, whereas the mean pore size and its distribution are key factors for a structure to have the highest possible specific surface area, which promotes the performance of a porous catalyst. The tortuosity and constrictivity of the transport pathways are other significant factors for lowering the mass-transport limitations in open porous materials [30,31,32,33,34]. An illustrative example of the advantageous use of an open porous catalyst is as used in the cathode of a molten carbonate fuel cell (MCFC). It has also been previously shown that the distribution of pore size in open porous materials strongly influences pressure drop behavior [32]. 

Porous catalysts or catalysts supported by porous materials are attracting great attention in the area of processes related to chemical synthesis, transformation to value-added products (especially fuels) and the mineralization of organic compounds. One of the processes with great ecological and industrial importance is the Sabatier reaction, which involves the reaction of hydrogen with carbon oxide CO*_x_* (x = 1, 2) at elevated temperatures and pressures over a catalyst towards production of methane and water [35,36]. The Sabatier process exhibits several similarities to the operation of MCFCs since both involve carbon dioxide as the main reagent and nickel as the catalyst. Our studies have shown that a specific microstructural design of the porous material for the cathode of high-temperature fuel cells can double the catalytic efficiency [37].

There are a few publications showing the successful incorporation of 3D printing FDM/FFF (Fused Filament Fabrication) in the preparation of metallic/ceramic systems based on thermoplastic polymers, e.g., alumina in PVB (polyvinyl butyral) and polyethyleneglycol (as plasticizer) [38], nickel powder in ABS matrix [39], silver powder with saturated polyester and polyvinyl butyral [40] and flaky silver powder in PVB [41], but this area of research is still at an early stage and no systematic investigations of Ni–polymer composite filaments are presented.

**Figure 1:**
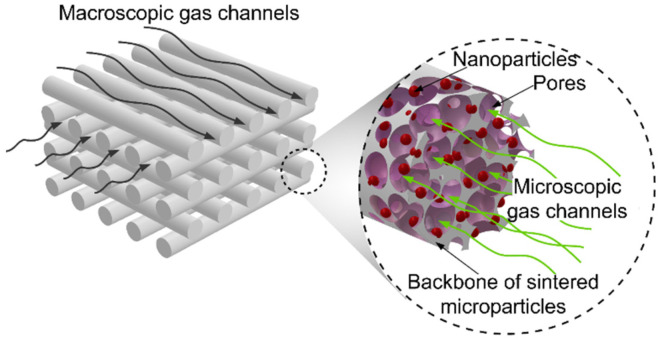
Schematic illustration of the possible hierarchical open porous microstructure of catalytic materials.

In summary, the incorporation of 3D printing methods in manufacturing processes provides new possibilities for the design of complex, hierarchical porous structures for catalysts in order to promote and facilitate optimal reaction pathways (Figure 1). There is now some maturity in this field, but further research is still warranted, and it remains a scientifically and industrially relevant research area. While the 3D printing of polymeric materials using this technique is now common [42], the printing of metallic materials is in the developmental stage. One of the main problems is the production of composite filaments and methods of further thermochemical processing [43,44]. An additional step might be, for instance, the post fabrication modification of the porous structure with metallic nanoparticles or other materials that enhance catalytic properties.

Our research goal was to design novel open porous catalyst structures using a sophisticated 3D printing technique. The research consisted of the development and use of functional 3D printing composite filaments, where the catalyst substrate (nickel powder) was embedded into a polymeric binder (PLA, PVB or ABS). Nickel was used because of its optimal properties for high-temperature catalysis and electrocatalysis, while it also has acceptable cost and availability for such applications. PLA, PVB and ABS have all been used successfully as filament materials in 3D printing using FDM. PLA has the advantage that it can be easily melted, while PVB and ABS have relatively high strength and plasticity, and dissolve in acetone, making them natural choices for testing filaments and porous materials using FDM. Filaments with a diameter of 2.85 mm were extruded from the composite materials, and then 3D printing was carried out using the Fused Deposition Modeling method. The 3D printed parts were thermally treated in order to remove the organic constituents and to partly sinter the metal particles. This produced the desired highly porous structure. The composite filaments and final materials were characterized by SEM (scanning electron microscopy) to describe the surface morphology of the materials, were analyzed with EDS and XRD to determine the chemical composition and they also underwent open porosity testing using the fluid saturation method based on Archimedes’ principle.

## 2. Materials and Methods

### 2.1. Materials

The polymers used belong to different chemical compound groups but are all thermoplastics, namely: polylactic acid (PLA Granulate, 3devo B.V., Utrecht, The Netherlands), acrylonitrile butadiene styrene (ABS Granulate, 3devo B.V., Utrecht, The Netherlands) and polyvinyl butyral (PVB Granulate, Mowital G36, Kuraray Europe GmbH, Hattersheim, Germany). Dissolution acetone (C_3_H_6_O, analytical grade, P.P.H. STANLAB, Lublin, Poland) was used as the solvent for PVB and ABS. Nickel powder (Ni, >99.7 wt%, T255™, Premium Grade, Vale, Toronto, ON, Canada) of high purity with a fine, three-dimensional structure was chosen as the filler. The diameter of nickel powder particles used was 2.2–2.6 μm, but the particles typically agglomerate into bigger ‘chain-like’ structures. All the reagents were used as received without any further purification. Additionally, nickel foam (Gelon Lib Co. Ltd., Shandong, China) of 0.5 mm thickness, surface density of 250 g/m^2^ and pore packing density of 100 ppi was used as a support for the 3D printed materials. Such support significantly increases mechanical strength of the element (see [45]).

### 2.2. Composite Preparation

The preparation procedure depended on the polymer used: for PLA a melting method was applied and for PVB and ABS, a solvent assisted method was used.

- *PLA-Ni Composite Preparation*

Polymer granulate was weighed and then melted in a ceramic mortar heated on a heating plate at a temperature of about 200 °C. After it was completely melted, nickel powder was added. The polymer to nickel weight ratio was controlled to obtain 5%, 10% and 20% volume fractions of nickel in the final composite material (see Table 1). The blend was thoroughly mixed with a spatula until a homogeneous consistency was obtained. The mixture was then thoroughly spread over a foil substrate and allowed to harden completely. After a few hours, the composite obtained was stripped from the foil and cut into granules (approximately 5 × 5 mm) suitable for the filament extrusion process.

- *ABS-Ni and PVB-Ni Composites Preparation*

ABS or PVB granules were weighed in a container and then acetone was added as a solvent. The mixture was stirred vigorously with a laboratory spoon and then placed in a THINKY mixer (Planetary Centrifugal Vacuum Mixer, model: ARV-930 TWIN; THINKY U.S.A., INC, Laguna Hills, CA, USA) until the polymer was completely dissolved (mixing parameters: revolution 800 rpm, vacuum 90 kPa, time 10 min). A strictly defined amount of nickel powder was added to the mixture (see Table 1). The mixture was remixed until a smooth homogeneous consistency was obtained. The slurries were poured over the foil and spread evenly. They were left for 2–3 days to allow for the complete evaporation of the solvent. After this time, the composites were stripped from the foil and then cut into fine granules (approximately 5 × 5 mm).

In this way, different composite materials with two different polymer matrices and different amounts of nickel powder were prepared with 10%, 15%, 20%, 25% and 50% by volume of filler for the PVB-Ni composite and 10%, 20%, 25% and 50% for the ABS-Ni composite. The composite granules obtained with the procedure described in Section 2.2. were thoroughly dried before extrusion and stored with a desiccant. 

### 2.3. Composite Filament Extrusion

**Figure 2:**
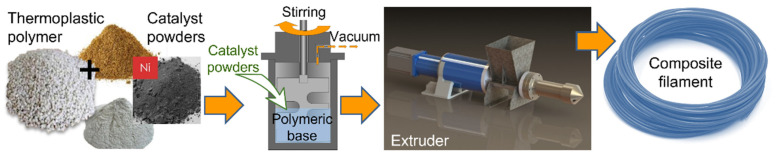
Fabrication route of new composite filaments.

A Precision 350 extruder (3devo B.V., Utrecht, The Netherlands) was used to fabricate filaments suitable for 3D FDM printing. The individual heating zones were set according to Table 2, from the screw to the nozzle. The extruder was first purged with commercially available pellets of the pure polymer corresponding to the composite. Subsequently, the fragmented composite was placed in the extruder tray and both the temperature, and the extrusion speed were controlled throughout the process (Table 2) to obtain a filament of the desired thickness (diameter of 2.85 mm). The screw rotation speed was adjustable from 2 to 5 rpm. The uniformity of the extruded filament was monitored on an ongoing basis. After extrusion, the filament was stored with a desiccant to prevent any moisture absorption. The procedure for the fabrication of composite filaments is schematically illustrated in Figure 2.

### 2.4. 3D Printing

The polymer–Ni composite filaments fabricated within these studies were used as the printing materials via FDM method. The parts were prepared using the Ultimaker S5 printer (Ultimaker B.V., Utrecht, The Netherlands). A module for printing materials containing metallic powders was used for the research (nozzle head CC 0.6). The printing platform was equipped with a heating system. Before printing, a 3D model was prepared in a format supported by the printer (.stl) in Cura 4.7.1 software (Ultimaker B.V., Utrecht, The Netherlands) compatible with the printer. For a given model, printing parameters were introduced, and then the model was cut into layers (slicing). The file obtained was entered into the printing program. 3D printing was carried out in two ways: on a nickel foam support and without it. The printing parameters presented in Table 3 have been precisely matched to the composite material. Printing was carried out only for composite filaments suited to this method.

### 2.5. Thermal Treatment

**Figure 3:**
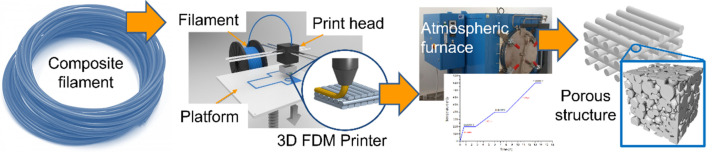
Fabrication of open porous materials by application of 3D printing.

After printing and prior to sintering, organic moieties have to be removed from the composite printed parts by debinding with a thermal treatment according to a procedure optimized in previous studies [46]. For this purpose, a Czylok FCF-V70CR retort furnace (Czylok, Jastrzębie-Zdrój, Poland) was used. Briefly, the samples were placed between two ceramic (alumina) setters, the thickness of which was 1.6 mm. The heat treatment was started by heating the furnace to a temperature of 200 °C. The heating rate was 5 °C/min. In order to remove volatile substances, after heating the furnace to 200 °C, a two-hour temperature step was used. The process was carried out in an air atmosphere. The oven was then heated to 400 °C at a rate of 1 °C/min. The heating process was carried out in a reducing atmosphere (95% N_2_ + 5% H_2_). The conditions were held for 2 h to burn off the polymeric binders. Finally, the oven was heated to 800 °C at a rate of 1 °C/min. A one-hour temperature step was used, again under a reducing atmosphere. This time was intended to thoroughly sinter the metallic particles resulting in a material with a porous structure. The use of a reducing atmosphere was to prevent nickel from oxidation during the thermal treatment process. The fabrication of open porous materials from 3D printing to final structure is shown in the Figure 3.

### 2.6. Characterization of Materials

Scanning electron microscopy (Hitachi TM-1000 Tabletop Microscope, Hitachi, Tokyo, Japan) was used to analyze the surface of the materials produced. Observations of the microstructure of composite filaments and 3D printed parts were carried out with an electron beam accelerating voltage of 15 kV. Composite filaments and corresponding 3D printed parts were tested after heat treatment. The analysis was performed to observe the surface morphology and to assess the distribution of nickel particles in the polymer matrix. Filament specimens were cut, and the cross-section was observed. The metallographic specimens were prepared with the use of a chemically hardened resin with graphite filler (Cold Mounting Material KEM 70, ATM Qness GmbH, Mammelzen, Germany) and surface grinding was performed on a manual grinder–polisher (SAPHIR 250 M2, ATM GmbH, Mammelzen, Germany). In order to determine the volume fraction of nickel particles in composites, computer image analysis (MicroMeter 1.0, Warsaw University of Technology, Warsaw, Poland) of the microscopic images was performed [47]. 

The elemental analysis of samples was performed via SEM microscopy using Phenom Pro X (ThermoFisher Scientific Phenom ProX, Waltham, MA, USA), FEI with an energy-dispersive X-ray detector (EDS). To reveal material composition, a backscatter electron (BSD) detector was used. Parameters used for all samples were as follows: elemental point analysis, images magnification in the range 5–8 k and voltage 15 kV.

The phase analysis of investigated materials after heat treatment was performed via X-ray diffraction (XRD) at room temperature using a Bruker D8 Advance (Bruker, Billerica, MA, USA) diffractometer with filtered CuKα (λ = 0.154056 nm) radiation. Running parameters were as follows: voltage 40 kV, current 40 mA, angular range 10° to 120°, step Δ2Θ − 0.05° and counting time 3 s. XRD patterns were analyzed using Bruker EVA software and a PDF-2 database (from the International Centre for Diffraction Data).

In order to analyze the porosity of the final materials, a hydrostatic weighing method based on Archimedes’ principle was applied. Measurements were carried out with the use of a kit for determining the density of solids and liquids KIT85 (RADWAG, Radom, Poland). Measurements were obtained of the 3D printed parts after firing, as well as of the unheated nickel foam and foam after firing. The total porosity, open porosity and closed porosity were calculated.

## 3. Results and Discussion

**Figure 4:**
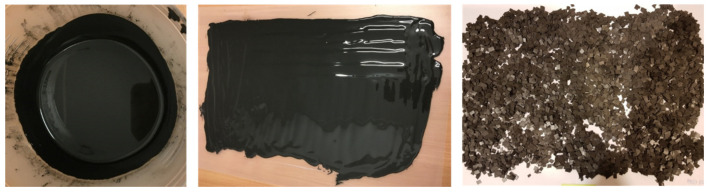
Composites at subsequent stages of fabrication: mixture of polymer, solvent and nickel powder after stirring (**left**), slurry after pouring on the foil (**middle**), final composite cut into granules (**right**).

The thermoplasticity of the material is particularly important to transform the material into a semi-liquid form for the methods and temperatures used for filament preparation and 3D printing. Thus, only thermoplastic polymers were selected for the research. Nickel powder was used as a filler since it is a widely used material in high-temperature catalysis and electrocatalysis (e.g., fuel cell components [35,45,46]). Preliminary research carried out as a part of this study concerned the selection of an appropriate solvent for PVB and ABS polymers. Cyclohexanone was used at first due to previous research experience with this solvent in the production of catalytic materials [46]. Both polymers dissolve in cyclohexanone but despite the long drying of the shredded composite in the dryer, the intense smell of cyclohexanone was still present, which indicated the presence of residual solvent in the material. Additionally, its presence in the composite during the extrusion process hindered the production of the filament. Hence, it was decided to choose acetone as a solvent for the other samples. It has a lower boiling point (56.2 °C) than cyclohexanone (154.3 °C), which greatly facilitates the drying process. For all composites that were prepared, the material appeared as presented in Figure 4, regardless of the solvent used. In the case of PLA-based composites, the only difference was at the beginning when the slurry had the consistency of a paste due to the melting technique used.

The slurry was uniformly mixed with a planetary mixer, giving a smooth surface with no air bubbles present. The composites dried in 2–3 days and then they were cut into small pieces. The granules obtained were stable and crumbled slightly during grinding but produced similar sized elements (approximately 5 × 5 mm).

The quality of the extrusion process is affected by the presence of metallic particles in the composite as confirmed by our experiments. First, it was observed that the greater the amount of additives in the polymer, the more difficult the extrusion process was. Depending on the base polymer, the temperatures were initially selected for the polymer, and then adapted to the extruded material by increasing or lowering the temperatures of the individual heating zones. It is important that the composite is well dried. Attempts to produce PVB and ABS composites using cyclohexanone as a solvent, or a mixture of cyclohexanone and acetone did not allow for the effective production of filaments. The presence of even residual amounts of cyclohexanone in the composite negatively influenced the process, resulting in the filament’s inhomogeneity, its breaking and even clogging of the device nozzle. Acetone has a low boiling point; hence, it evaporates easily, and the composites obtained during the tests were properly dried.

**Figure 5:**
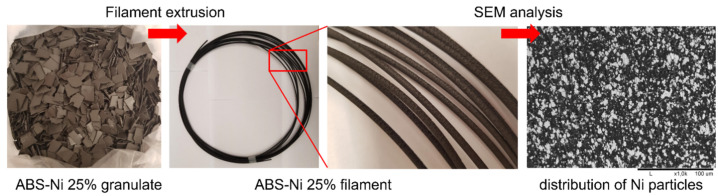
ABS-Ni 25% composite granulate and obtained filament. SEM image of the cross-section of the filament showing the distribution of nickel particles in the polymer matrix.

Each composite required its own extrusion process parameters, such as the temperatures of individual heating zones, from the screw to the nozzle (Table 2), the speed of rotation of the screw (average 4 rpm), and thus the speed of extrusion. All this influenced the final quality of the product, especially the thickness of the filament, its homogeneity and continuity. Too high a nickel content made it impossible to extrude the filaments. In the case of the PLA-based composite, this was for a nickel content above 5–10% by volume; for PVB and ABS, it was for a nickel content over 25%. All successfully produced filaments had a diameter in the range of 2.70–3.00 mm, so they were suitable for FDM 3D printing (see Table 4). All results presented in the table for PVB and ABS-based composites were obtained for samples with acetone. Importantly, they did not tear during manufacturing or crack afterwards and were stiff enough to be used in a 3D printer. As an illustration of the extrusion process, and showing the extruded composite filament, the results for the ABS-Ni 25% composite are presented in Figure 5.

**Figure 6:**
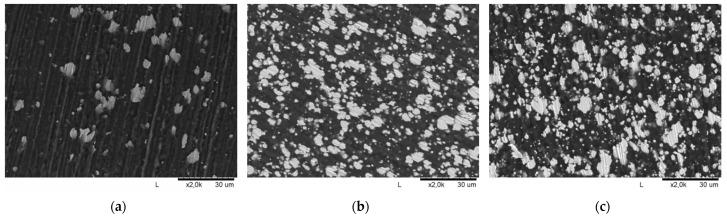
SEM images of the composite filaments: (**a**) PLA-Ni 5%; (**b**) PVB-Ni 25%; (**c**) ABS-Ni 25%.

All filaments, that were deemed to have a high potential to be useful, were subjected to SEM microscopic analysis (Figure 6). SEM imaging confirmed the uniform distribution of nickel particles in the polymer matrices and allowed for the determination of the volume fraction of nickel in the composites. As shown in Figure 6, the powder particles tend to form agglomerates due to the mutual adherence of particles to each other, but their distribution as illustrated in the cross-sectional images of the filament is homogeneous and regular. Computer image analysis performed by MicroMeter 1.0 software confirmed the effectiveness of the production of composites with the previously chosen volume fractions of nickel. For PLA-Ni 5%, it was 6 ± 1 vol%; for PVB-Ni 25%, it was 29 ± 3 vol%; and for ABS-Ni 25%, it was 26 ± 2 vol%. This slight difference may be due to the loss of polymer material during the production of composites (polymer deposition on the walls of the container and spatula). A uniform distribution of particles in the matrix was successfully obtained, which is essential for 3D printing.

**Figure 7:**
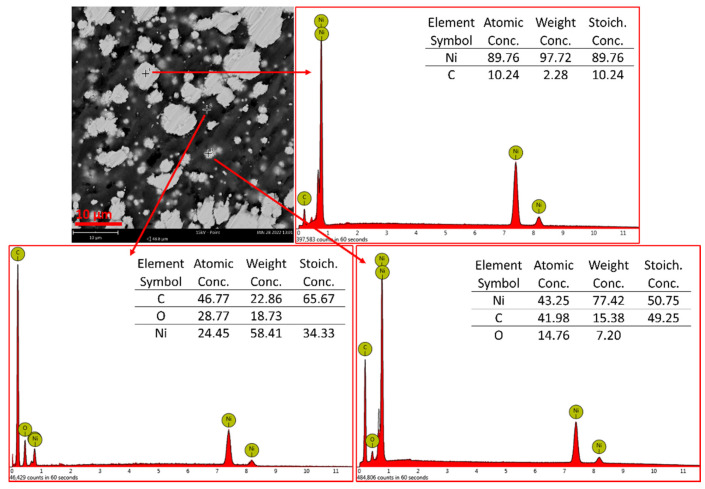
SEM image and representative EDS spectra with element concentrations for composite filament cross-section performed for different points on the sample (PVB-Ni 25%).

The analysis of composite materials was performed via SEM microscopy equipped with energy-dispersive X-ray detector and confirmed the presence of three elements in the samples: Ni, C and O. Figure 7 shows the SEM image of composite filament cross-section of PVB-Ni 25% and the corresponding EDS point analysis. Brighter spots are nickel particles and darker areas the polymer. Characteristic energy spectral lines for nickel can be seen (7.48 keV for Kα, 0.85 keV for Lα). 

The above-mentioned composite filaments were selected for 3D printing. All polymers selected had a good adhesion to the printer’s working glass substrate. For some of the samples, nickel foam was used as the substrate to improve the mechanical properties, and more specifically, the fracture toughness of the final material [45].

**Figure 8:**
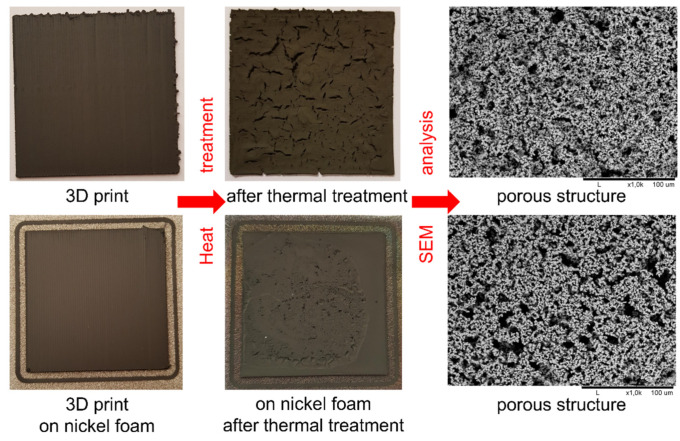
3D printed sample using the ABS-Ni 25% filament (**upper images**) and similar sample printed on nickel foam (**lower images**) with images of samples after heat treatment and the corresponding SEM images showing the porous structures.

**Figure 9:**
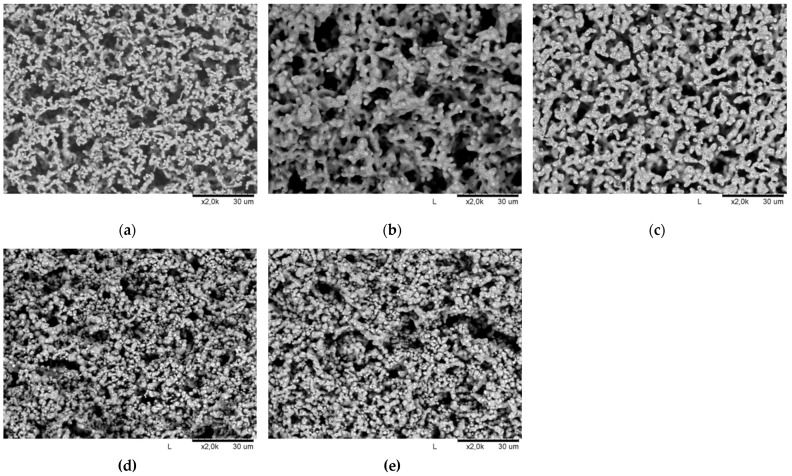
SEM images of the printout morphology after heat treatment: (**a**) PLA-Ni 5% on Ni foam; (**b**) PVB-Ni 25% and (**c**) on Ni foam; and (**d**) ABS-Ni 25% and (**e**) on Ni foam.

As shown in Figure 8, the 3D printed parts were homogeneous, without any significant defects. After heat treatment, the 3D printed parts retained their dimensions, regardless of the presence or absence of nickel foam. During the printing process itself, the composite material partially penetrates the top part of the foam, which affected the process of sample burning in the oven. The material was partially cracked as a result of material shrinkage. Figure 9 presents SEM images of the morphology of the printed parts after heat treatment: PLA-Ni 5% on Ni foam (a), PVB-Ni 25% (b) and on Ni foam (c), and ABS-Ni 25% (d) and on Ni foam (e). It can be seen that a uniform particle size resulted in controlled porosity in the sintered 3D printed parts for all tested samples. It can be concluded that the materials produced are characterized by an open pore structure. Nevertheless, traces of unremoved polymer are still visible on the nickel particles.

**Figure 10:**
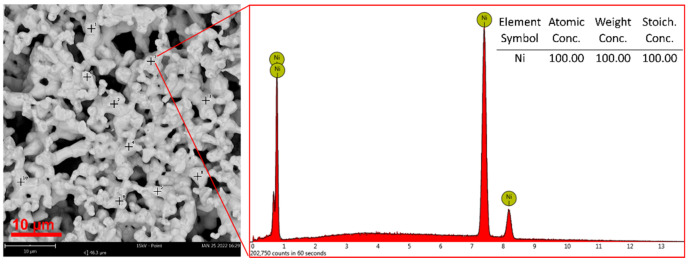
SEM image and representative EDS spectrum for PVB-Ni 25% print after heat treatment.

The porous structure of the PVB-Ni 25% print after heat treatment can be seen in the SEM image (Figure 10). Sintered nickel particles are clearly visible. The elemental analysis confirmed that the obtained materials are made of metallic nickel. The presence of oxygen was not observed in the spectra. Similar results were obtained for all tested materials. Additionally, effectiveness of the pure nickel material preparation was also subsequently confirmed by the XRD analysis.

**Figure 11:**
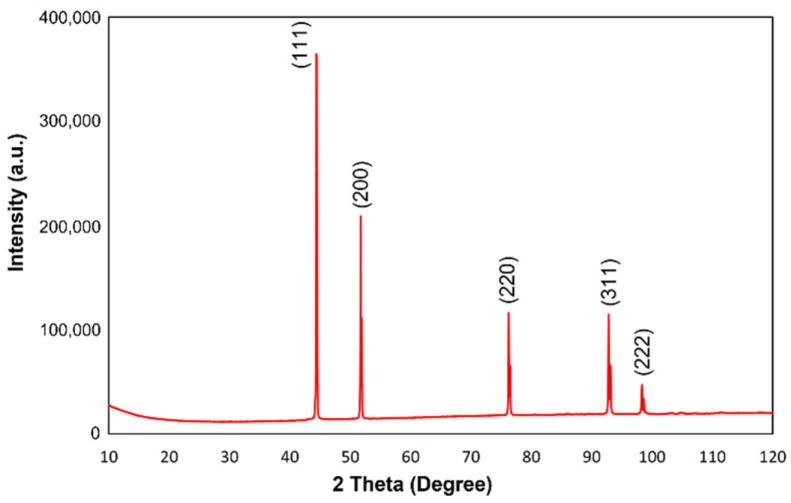
XRD pattern for nickel porous structure.

The phase analysis of investigated materials after heat treatment performed via X-ray diffraction confirmed the presence of pure nickel in the samples. Figure 11 depicts the XRD pattern of Ni with five diffraction peaks: 111, 200, 220, 311 and 222, which are comparable with the standard powder diffraction card (JCPDS card, no. 04-0850). No peaks corresponding to nickel oxide were observed (JCPDS card, no. 47-1049 for nickel oxide). As a result of the analysis, the effectiveness of the applied heat treatment procedure was confirmed, as well as the use of the reducing gas mixture during the process, which led to the protection of nickel against oxidation.

Table 5 presents selected physical properties of the porous materials produced, as determined by the Archimedes method. When comparing the results, it should be borne in mind that the values obtained for the samples printed on the foam substrate are functions of the porosity of the foam and the material. Moreover, it is difficult to estimate to what extent the printed material fused into the substrate after the firing process, which makes it impossible to calculate individual components. The determination of some physical properties, materials with an open cell structure, also has an error related to the leakage of water from the pores during mass measurements.

The results show that all the tested samples, obtained from the printed composite filaments were characterized by a high total porosity of above 60%. As can be seen, the porosity of the foam decreased slightly after the firing process with a small increase in the closed porosity, although it is within the measurement error. The use of a support in the form of nickel foam in the three-dimensional printing process increases the total porosity of the polymer–Ni composite material on the other hand after thermal treatment. The average open porosity of the material also increased due to the high porosity of the substrate. All the samples printed on the nickel foam had an open porosity parameter higher than 68% which is very promising for further research. Open porosity is crucial for the efficient transport of catalytic reactants (gas or liquid) in the material. The analysis of the results obtained for closed porosity shows that materials printed without foam had the highest closed porosity value–slightly over 5%, and the smallest one printed with PVB-Ni 25% on a foam substrate. It can be seen that the use of a nickel foam substrate allows for a greater total and open porosity for the same type of filament, which is advantageous for catalysis.

## 4. Summary and Conclusions

This article presents the development of different types of composite materials using a thermoplastic polymer as matrix and nickel powder as filler. The composites differed in the type of polymer used (PLA, PVB or ABS) and the volume fraction of nickel (in the range of 5 to 50 vol%). Depending on the nickel content in the composite and the polymer used, some of the composites were not suitable for the extrusion of filaments. The best results were obtained for the following composites: PLA-Ni 5%, PVB-Ni 25% and ABS-Ni 25%. Filaments extruded from these materials were characterized by homogeneity of the material in terms of the distribution of nickel particles in the composite and homogeneity of the filament diameter; the materials did not break or crumble, and at the same time were stiff enough to effectively print. SEM microscopic analysis of the cross-sections of the extruded filaments showed the morphology of the samples as well as the uniform distribution of nickel particles in the polymer matrix. Computer analysis of the images confirmed the volume fraction of nickel in the composites arranged during mixing. EDS elemental analysis confirmed the materials composition and the XRD measurements verified the pure nickel presence in the final porous structure.

Composite filaments were used as printing materials in the FDM method. 3D printing was carried out in two ways: on a nickel foam substrate and without the use of a substrate. The surface of the 3D printed parts was characterized by a smooth surface without lumps and unevenness. The printed parts did not crumble or deform. They were subsequently subjected to heat treatment processes in order to remove the polymer matrix, sinter the nickel particles and to obtain open porous materials. SEM analysis and porosity measurements confirmed uniform morphology and a highly open porous structure, generally above 60%, which makes them suitable for further catalytical tests. The use of nickel foam in 3D printing had a double function. Firstly, it increased the durability of the printed parts after heat treatment, and it also increased the porosity of the final material.

The results of the study allow us to formulate the following conclusions:

1. Complete removal of the solvent in the drying process of composites is a crucial condition for proper filament extrusion and for obtaining a material useful in 3D printing with FDM.
2. The use of acetone as a solvent in PVB and ABS based composites made it possible to carry out the process of drying and filament extrusion with an appropriate content of nickel particles.
3. As the nickel content increases, the brittleness of the polymer-Ni filament increases. With a volume content of Ni above 25% for the solvent-based composite preparation method, the plasticity of the material decreases, which makes it almost impossible to be printed with FDM. For the PLA-based composite, the polymer melting method works only for nickel contents, at about 5%. At higher ones, the material is too brittle.
4. It has been shown that PLA-Ni 5%, PVB-Ni 25% and ABS-Ni 25% composite filaments allow for the 3D printing of parts with a flat geometry, which after appropriate thermal treatment led to a metallic structure with an open porosity above 60%, while maintaining satisfactory mechanical properties.

In further research, it is proposed that the composition of the composites is modified to enable the 3D printing of structures with more complex spatial geometries. Concurrently, catalytical tests of the developed materials will be carried out for a specific application.

## Figures and Tables

**Table 1:** Substrates amount used for composite preparation; all samples prepared with 100 g of nickel powder.

Polymer Used	Ni Volume Fraction in a Composite (vol%)	Polymer Amount (g)
PLA	5	264.7
	10	125.4
	20	55.7
PVB	10	111.31
	15	70.04
	20	49.58
	25	37.09
	50	12.37
ABS	10	105.19
	20	46.82
	25	35.03

**Table 2:** Composite filament extrusion temperatures and speeds for selected composite materials; heating zones are numbered from screw (1) to nozzle (4).

	PLA-Ni 5%	PVB-Ni 25%	ABS-Ni 25%
Heaters	Set temperature (℃)		
4	170	170	220
3	185	195	230
2	190	200	230
1	180	200	240
	Average extrusion speed (rpm)		
	~4.0	~3.0	~3.5

**Table 3:** FDM printing parameters for the selected feedstock systems.

Item	PLA-Ni 5%	PVB-Ni 25%	ABS-Ni 25%
Printing temperature	220 °C	230 °C	250 °C
Printing bed temperature	65 °C	85 °C	85 °C
Printing speed	15 mm/s		
Single layer height	0.4 mm		
Line width	0.4 mm		
Print dimensions	5 cm × 5 cm × 0.12 cm		
Infill Line Directions	90 degrees		

**Table 4:** Prepared composites and their corresponding filaments.

Ni Powder vol% in Composite	PLA-Ni	PVB-Ni	ABS-Ni
**50%**	-	unsuitable for extrusion (89 wt% of Ni)	-
**25%**	-	+ (73 wt% of Ni)	+ (74 wt% of Ni)
**20%**	unsuitable for extrusion (64 wt% of Ni)	+ (67 wt% of Ni)	+ (68 wt% of Ni)
**15%**	-	+ (59 wt% of Ni)	-
**10%**	filament too brittle, it breaks (44 wt% of Ni)	+ (47 wt% of Ni)	+ (49 wt% of Ni)
**5%**	+ (27% wt% of Ni)	-	-

- no attempt made; + obtained filament was suitable for 3D printing.

**Table 5:** Calculation results of porosity of nickel foam and final 3D prints.

Average Parameter	Ni Foam	Thermally Treated Ni Foam	PLA-Ni 5% on Ni Foam	PVB-Ni 25%		ABS-Ni 25%	
				-	on Ni Foam	-	on Ni Foam
Total porosity (%)	90.9	86.1	86.6	60.7	71.8	65.7	76.3
Open porosity (%)	90.8	85.5	84.1	55.2	68.3	59.8	71.6
Closed porosity (%)	0.1	0.6	2.4	5.4	3.5	5.9	4.7

## Data Availability

All data are available from authors after a reasonable request.
